# On the Depth of Decision Trees with Hypotheses

**DOI:** 10.3390/e24010116

**Published:** 2022-01-12

**Authors:** Mikhail Moshkov

**Affiliations:** Computer, Electrical and Mathematical Sciences & Engineering Division, King Abdullah University of Science and Technology (KAUST), Thuwal 23955-6900, Saudi Arabia; mikhail.moshkov@kaust.edu.sa

**Keywords:** test theory, rough set theory, exact learning, decision trees, complexity classes

## Abstract

In this paper, based on the results of rough set theory, test theory, and exact learning, we investigate decision trees over infinite sets of binary attributes represented as infinite binary information systems. We define the notion of a problem over an information system and study three functions of the Shannon type, which characterize the dependence in the worst case of the minimum depth of a decision tree solving a problem on the number of attributes in the problem description. The considered three functions correspond to (i) decision trees using attributes, (ii) decision trees using hypotheses (an analog of equivalence queries from exact learning), and (iii) decision trees using both attributes and hypotheses. The first function has two possible types of behavior: logarithmic and linear (this result follows from more general results published by the author earlier). The second and the third functions have three possible types of behavior: constant, logarithmic, and linear (these results were published by the author earlier without proofs that are given in the present paper). Based on the obtained results, we divided the set of all infinite binary information systems into four complexity classes. In each class, the type of behavior for each of the considered three functions does not change.

## 1. Introduction

Decision trees are studied in different areas of computer science, in particular in exact learning [1], rough set theory [2,3,4], and test theory [5]. In some sense, these theories deal with dual objects: for example, membership queries from exact learning correspond to attributes from test theory and rough set theory. In contrast to test theory and rough set theory, in exact learning, besides membership queries, equivalence queries are also considered.

We extend the model considered in test theory and rough set theory by adding the notion of a hypothesis that is an analog of equivalence query. Papers [6,7,8,9,10] are related mainly to the experimental study of decision trees with hypotheses. The present paper contains a theoretical study of the depth of decision trees with hypotheses.

An infinite binary information system is a pair U=(A,F) where *A* is an infinite set of elements and *F* is an infinite set of functions (attributes) from *A* to {0,1}. A problem over *U* is given by a finite number of attributes $f_{1},\ldots ,f_{n}$ from *F*: for a∈A, we should find the tuple $\left(f_{1}\left(a\right),\ldots ,f_{n}\left(a\right)\right)$. To solve this problem, we can use decision trees with two types of queries. We can ask about the value of an attribute $f_{i}\in \left\{f_{1},\ldots ,f_{n}\right\}$. As a result, we obtain an answer of the kind $f_{i}\left(x\right)=\delta$ where δ∈{0,1}. We also can ask if a hypothesis $f_{1}\left(x\right)=\delta_{1},\ldots ,f_{n}\left(x\right)=\delta_{n}$ is true where $\delta_{1},\ldots ,\delta_{n}\in \left\{0,1\right\}$. Either we obtain the confirmation or a counterexample in the form $f_{i}\left(x\right)=\neg \delta_{i}$.

The depth of decision trees with hypotheses can be essentially less than the depth of decision trees using only attributes. As an example, we consider the problem of the computation of the disjunction $x_{1}\vee \cdots \vee x_{n}$. The minimum depth of a decision tree solving this problem using only attributes $x_{1},\ldots ,x_{n}$ is equal to *n*. However, the minimum depth of a decision tree with hypotheses solving this problem is equal to one: it is enough to ask only about the hypothesis $x_{1}=0,\ldots ,x_{n}=0$. If it is true, then the considered disjunction is equal to zero. Otherwise, it is equal to one.

Based on the results of exact learning, rough set theory, and test theory [1,11,12,13,14,15,16], we study for an arbitrary infinite binary information system three functions of the Shannon type that characterize the growth in the worth case of the minimum depth of a decision tree solving a problem with the growth of the number of attributes in the problem description. The considered three functions correspond to the following three cases:

(i) Only attributes are used in decision trees;
(ii) Only hypotheses are used in decision trees;
(iii) Both attributes and hypotheses are used in decision trees.

We show that the first function has two possible types of behavior: logarithmic and linear. The second and third functions have three possible types of behavior: constant, logarithmic, and linear. Bounds for the case (i) can be derived from more general results obtained in [15,16]. Results related to the cases (ii) and (iii) were presented in the conference paper [17] without proofs. In the present paper, we consider complete proofs for the cases (ii) and (iii). We also investigate the join behavior of these three functions and describe four complexity classes of infinite binary information systems; these results are completely new.

The obtained results allow us to understand the difference of time complexity for conventional decision trees that use only queries based on one attribute each and for decision trees with hypotheses. Moreover, we know now which combinations of types of behavior of the three Shannon-type functions we can take under consideration of an arbitrary infinite binary system, and we know the criteria for each combination.

This paper consists of six sections. In Section 2 and Section 3, we consider the basic notions and main results. Section 4 and Section 5 contain proofs of the main results, and Section 6 gives a short conclusion.

## 2. Basic Notions

Let *A* be a set of elements and *F* be a set of functions from *A* to {0,1}. Functions from *F* are called *attributes*, and the pair U=(A,F) is called a *binary information system* (this notion is close to the notion of information systems proposed by Pawlak [18]). If *A* and *F* are infinite sets, then the pair U=(A,F) is called an *infinite binary information system*.

A *problem over* *U* is an arbitrary *n*-tuple $z=(f_{1},\ldots ,f_{n})$ where n∈N, N is the set of natural numbers {1,2,…}, and $f_{1},\ldots ,f_{n}\in F$. The problem *z* may be interpreted as a problem of searching for the tuple $z\left(a\right)=(f_{1}\left(a\right),\ldots ,f_{n}\left(a\right))$ for an arbitrary a∈A. The number dimz=n is called the *dimension* of the problem *z*. Denote $F\left(z\right)=\left\{f_{1},\ldots ,f_{n}\right\}$. We denote by P(U) the set of problems over *U*.

A *system of equations over U* is an arbitrary equation system of the kind:

$$\left\{g_{1}\left(x\right)=\delta_{1},\ldots ,g_{m}\left(x\right)=\delta_{m}\right\}$$

where m∈N∪{0}, $g_{1},\ldots ,g_{m}\in F$, and $\delta_{1},\ldots ,\delta_{m}\in \left\{0,1\right\}$ (if m=0, then the considered equation system is empty). This equation system is called a *system of equations over z* if $g_{1},\ldots ,g_{m}\in F\left(z\right)$. The considered equation system is called *consistent* (on *A*) if its set of solutions on *A* is nonempty. The set of solutions of the empty equation system coincides with *A*.

As algorithms for problem *z* solving, we consider decision trees with two types of *queries*. We can choose an attribute $f_{i}\in F\left(z\right)$ and ask about its value. This query has two possible answers: $\left\{f_{i}\left(x\right)=0\right\}$ and $\left\{f_{i}\left(x\right)=1\right\}$. We can formulate a *hypothesis over* *z* in the form $H=\{f_{1}\left(x\right)=\delta_{1},\ldots ,f_{n}\left(x\right)=\delta_{n}\}$ where $\delta_{1},\ldots ,\delta_{n}\in \left\{0,1\right\}$ and ask about this hypothesis. This query has n+1 possible answers: $H,\left\{f_{1}\left(x\right)=\neg \delta_{1}\right\},\ldots ,\left\{f_{n}\left(x\right)=\neg \delta_{n}\right\}$ where ¬1=0 and ¬0=1. The first answer means that the hypothesis is true. Other answers are counterexamples.

A *decision tree over* *z* is a marked finite directed tree with the root in which:

- Each *terminal* node is labeled with an *n*-tuple from the set ${\left\{0,1\right\}}^{n}$;
- Each node, which is not terminal (such nodes are called *working*), is labeled with an attribute from the set F(z) or with a hypothesis over *z*;
- If a working node is labeled with an attribute $f_{i}$ from F(z), then there are two edges, which leave this node and are labeled with the systems of equations $\left\{f_{i}\left(x\right)=0\right\}$ and $\left\{f_{i}\left(x\right)=1\right\}$, respectively;
- If a working node is labeled with a hypothesis:
  $$H=\{f_{1}\left(x\right)=\delta_{1},\ldots ,f_{n}\left(x\right)=\delta_{n}\}$$
  over *z*, then there are n+1 edges, which leave this node and are labeled with the system of equations $H,\left\{f_{1}\left(x\right)=\neg \delta_{1}\right\},\ldots ,\left\{f_{n}\left(x\right)=\neg \delta_{n}\right\}$, respectively.

Let Γ be a decision tree over *z*. A *complete path* in Γ is an arbitrary directed path from the root to a terminal node in Γ. We now define an equation system S(ξ) over *U* associated with the complete path ξ. If there are no working nodes in ξ, then S(ξ) is the empty system. Otherwise, S(ξ) is the union of equation systems assigned to the edges of the path ξ. We denote by A(ξ) the set of solutions on *A* of the system of equations S(ξ) (if this system is empty, then its solution set is equal to *A*).

We say that a decision tree Γ over *z* *solves the problem z relative to U* if, for each element a∈A and for each complete path ξ in Γ such that a∈A(ξ), the terminal node of the path ξ is labeled with the tuple z(a).

We now consider an equivalent definition of a decision tree solving a problem. Denote by $\Delta_{U}\left(z\right)$ the set of tuples $\left(\delta_{1},\ldots ,\delta_{n}\right)\in {\left\{0,1\right\}}^{n}$ such that the system of equations $\left\{f_{1}\left(x\right)=\delta_{1},\ldots ,f_{n}\left(x\right)=\delta_{n}\right\}$ is consistent. The set $\Delta_{U}\left(z\right)$ is the set of all possible solutions to the problem *z*. Let $\Delta \subseteq \Delta_{U}\left(z\right)$, $f_{i_{1}},\ldots ,f_{i_{m}}\in \left\{f_{1},\ldots ,f_{n}\right\}$, and $\sigma_{1},\ldots ,\sigma_{m}\in \left\{0,1\right\}$. Denote:

$$\Delta \left(f_{i_{1}},\sigma_{1}\right)\cdots \left(f_{i_{m}},\sigma_{m}\right)$$

the set of all *n*-tuples $(\delta_{1},\ldots ,\delta_{n})\in \Delta$ for which $\delta_{i_{1}}=\sigma_{1},\ldots ,\delta_{i_{m}}=\sigma_{m}$.

Let Γ be a decision tree over the problem *z*. We correspond to each complete path ξ in the tree Γ a word π(ξ) in the alphabet $\left\{\left(f_{i},\delta \right):f_{i}\in F\left(z\right),\delta \in \left\{0,1\right\}\right\}$. If the equation system S(ξ) is empty, then π(ξ) is the empty word. If $S\left(\xi \right)=\{f_{i_{1}}\left(x\right)=\sigma_{1},\ldots ,f_{i_{m}}\left(x\right)=\sigma_{m}\}$, then $\pi \left(\xi \right)=\left(f_{i_{1}},\sigma_{1}\right)\cdots \left(f_{i_{m}},\sigma_{m}\right)$. The decision tree Γ over *z* solves the problem *z* relative to *U* if, for each complete path ξ in Γ, the set $\Delta_{U}\left(z\right)\pi \left(\xi \right)$ contains at most one tuple, and if this set contains exactly one tuple, then the considered tuple is assigned to the terminal node of the path ξ.

As the time complexity of a decision tree Γ, we consider its *depth* h(Γ), that is the maximum number of working nodes in a complete path in the tree Γ.

Let z∈P(U). We denote by $h_{U}^{\left(1\right)}\left(z\right)$ the minimum depth of a decision tree over *z*, which solves *z* relative to *U* and uses only attributes from F(z). We denote by $h_{U}^{\left(2\right)}\left(z\right)$ the minimum depth of a decision tree over *z*, which solves *z* relative to *U* and uses only hypotheses over *z*. We denote by $h_{U}^{\left(3\right)}\left(z\right)$ the minimum depth of a decision tree over *z*, which solves *z* relative to *U* and uses both attributes from F(z) and hypotheses over *z*.

For i=1,2,3, we define a function of the Shannon type $h_{U}^{\left(i\right)}\left(n\right)$ that characterizes the dependence of $h_{U}^{\left(i\right)}\left(z\right)$ on dimz in the worst case. Let i∈{1,2,3} and n∈N. Then:

$$h_{U}^{\left(i\right)}\left(n\right)=\max\left\{h_{U}^{\left(i\right)}\left(z\right):z\in P\left(U\right),\dim z\leq n\right\}.$$

## 3. Main Results

Let U=(A,F) be an infinite binary information system and r∈N. The information system *U* is called *r*-*reduced* if, for each consistent on *A* system of equations over *U*, there exists a subsystem of this system that has the same set of solutions and contains at most *r* equations. We denote by R the set of infinite binary information systems each of which is *r*-*reduced* for some r∈N.

The next theorem follows from the results obtained in [15], where we considered closed classes of test tables (decision tables). It also follows from the results obtained in [16], where we considered the weighted depth of decision trees.

**Theorem** **1.** *Let U be an infinite binary information system. Then, the following statements hold:*

*(a)*  *If U∈R, then $h_{U}^{\left(1\right)}\left(n\right)=\Theta \left(\log n\right)$;*
*(b)*  *If U∉R, then $h_{U}^{\left(1\right)}\left(n\right)=n$ for any n∈N.*

A subset $\left\{f_{1},\ldots ,f_{m}\right\}$ of *F* is called *independent* if, for any $\delta_{1},\ldots ,\delta_{m}\in \left\{0,1\right\}$, the system of equations $\left\{f_{1}\left(x\right)=\delta_{1},\ldots ,f_{m}\left(x\right)=\delta_{m}\right\}$ is consistent on the set *A*. The empty set of attributes is independent by definition. We now define the *independence dimension* or *I*-*dimension*I(U) of the information system *U* (this notion is similar to the notion of the independence number of the family of sets considered by Naiman and Wynn in [19]). If, for each m∈N, the set *F* contains an independent subset of cardinality *m*, then I(U)=∞. Otherwise, I(U) is the maximum cardinality of an independent subset of the set *F*. We denote by D the set of infinite binary information systems with a finite independence dimension.

Let U=(A,F) be a binary information system, which is not necessarily infinite, f∈F, and δ∈{0,1}. Denote:

A(f,δ)={a:a∈A,f(a)=δ}.

We now define inductively the notion of a *k*-information system, k∈N∪{0}. The binary information system *U* is called a 0-information system if all attributes from *F* are constant on the set *A*. Let, for some k∈N∪{0}, the notion of a *m*-information system be defined for m=0,…,k. The binary information system *U* is called a (k+1)-information system if it is not a *m*-information system for m=0,…,k and, for any f∈F, there exist numbers δ∈{0,1} and m∈{0,…,k} such that the information system (A(f,δ),F) is a *m*-information system. It is easy to show by induction on *k* that if U=(A,F) is a *k*-information system, then $U^{\prime }=\left(A^{\prime },F\right)$, $A^{\prime }\subseteq A$, is a *l*-information system for some l≤k. We denote by C the set of infinite binary information systems for each of which there exists k∈N such that the considered system is a *k*-information system. The following theorem was presented in [17] without proof.

**Theorem** **2.** *Let U be an infinite binary information system. Then, the following statements hold:*

*(a)*  *If U∈C, then $h_{U}^{\left(2\right)}\left(n\right)=O\left(1\right)$ and $h_{U}^{\left(3\right)}\left(n\right)=O\left(1\right)$;*
*(b)*  *If U∈D∖C, then $h_{U}^{\left(2\right)}\left(n\right)=\Theta \left(\log n\right)$, $h_{U}^{\left(3\right)}\left(n\right)=\Omega \left(\frac{\log n}{\log\log n}\right)$, and $h_{U}^{\left(3\right)}\left(n\right)=O\left(\log n\right)$;*
*(c)*  *If U∉D, then $h_{U}^{\left(2\right)}\left(n\right)=n$ and $h_{U}^{\left(3\right)}\left(n\right)=n$ for any n∈N.*

Let *U* be an infinite binary information system. We now consider the join behavior of the functions $h_{U}^{\left(1\right)}\left(n\right)$, $h_{U}^{\left(2\right)}\left(n\right)$, and $h_{U}^{\left(3\right)}\left(n\right)$. It depends on the belonging of the information system *U* to the sets R, D, and C. We correspond to the information system *U* its *indicator vector* $ind\left(U\right)=\left(c_{1},c_{2},c_{3}\right)\in {\left\{0,1\right\}}^{3}$ in which $c_{1}=1$ if and only if U∈R, $c_{2}=1$ if and only if U∈D, and $c_{3}=1$ if and only if U∈C.

**Theorem** **3.** *For any infinite binary information system, its indicator vector coincides with one of the rows of Table 1. Each row of Table 1 is the indicator vector of some infinite binary information system.*

For i=1,2,3,4, we denote by $V_{i}$ the class of all infinite binary information systems, for which the indicator vector coincides with the *i*th row of Table 1. Table 2 summarizes Theorems 1–3. The first column contains the name of *complexity class* $V_{i}$. The next three columns describe the indicator vector of information systems from this class. The last three columns $h_{U}^{\left(1\right)}\left(n\right)$, $h_{U}^{\left(2\right)}\left(n\right)$, and $h_{U}^{\left(3\right)}\left(n\right)$ contain information about the behavior of the functions $h_{U}^{\left(1\right)}\left(n\right)$, $h_{U}^{\left(2\right)}\left(n\right)$, and $h_{U}^{\left(3\right)}\left(n\right)$ for information systems from the class $V_{i}$.

## 4. Proof of Theorem 2

We precede with the proof of Theorem 2 by two lemmas.

Let d∈N. A *d*-*complete tree over the information system* U=(A,F) is a marked finite directed tree with the root in which:

- Each terminal node is not labeled;
- Each nonterminal node is labeled with an attribute f∈F. There are two edges leaving this node that are labeled with the systems of equations {f(x)=0} and {f(x)=1}, respectively;
- The length of each complete path (the path from the root to a terminal node) is equal to *d*;
- For each complete path ξ, the equation system S(ξ), which is the union of equation systems assigned to the edges of the path ξ, is consistent.

Let *G* be a *d*-complete tree over *U* and F(G) be the set of all attributes attached to the nonterminal nodes of the tree *G*. The number of nonterminal nodes in *G* is equal to $2^{0}+2^{1}+\ldots +2^{d-1}=2^{d}-1$. Therefore, $\left|F\left(G\right)\right|\leq 2^{d}$.

The results mentioned in the following lemma are obtained by methods similar to those used by Littlestone [12], Maass and Turán [13], and Angluin [11].

**Lemma** **1.** *Let U=(A,F) be a binary information system, d∈N, G be a d-complete tree over U, and z be a problem over U such that F(G)⊆F(z). Then*

*(a)*  *$h_{U}^{\left(2\right)}\left(z\right)\geq d$;*
*(b)*  *$h_{U}^{\left(3\right)}\left(z\right)\geq \frac{d}{\log_{2}\left(2d\right)}$.*

**Proof.**  (a) We prove the inequality $h_{U}^{\left(2\right)}\left(z\right)\geq d$ by induction on *d*. Let d=1. Then, the tree *G* has the only one nonterminal node, which is labeled with an attribute *f* that is not constant on *A*. Therefore, $|\Delta_{U}\left(z\right)|\geq 2$ and $h_{U}^{\left(2\right)}\left(z\right)\geq 1$. Let, for t∈N and for any natural *d*, 1≤d≤t, the considered statement hold. Assume now that d=t+1, *G* is a *d*-complete tree over *U*, z is a problem over *U* such that F(G)⊆F(z), and Γ is a decision tree over *z* with the minimum depth, which solves the problem *z* and uses only hypotheses. Let *f* be the attribute attached to the root of the tree *G* and *H* be the hypothesis attached to the root of the decision tree Γ. Then, there is an edge that leaves the root of Γ and is labeled with the equation system {f(x)=δ} where the equation f(x)=¬δ belongs to the hypothesis *H*. This edge enters to the root of the subtree of Γ, which is denoted by $\Gamma_{f}$. There is an edge that leaves the root of *G* and is labeled with the equation system {f(x)=δ}. This edge enters the root of the subtree of *G*, which is denoted by $G_{\delta }$. One can show that the decision tree $\Gamma_{f}$ solves the problem *z* relative to the information system $U^{\prime }=\left(A\left(f,\delta \right),F\right)$ and $G_{\delta }$ is a *t*-complete tree over $U^{\prime }$. It is clear that $F\left(G_{\delta }\right)\subseteq F\left(z\right)$. Using the inductive hypothesis, we obtain $h(\Gamma_{f})\geq t$. Therefore, h(Γ)≥t+1=d and $h_{U}^{\left(2\right)}\left(z\right)\geq d$.

(b) We now prove the inequality $h_{U}^{\left(3\right)}\left(z\right)\geq \frac{d}{\log_{2}\left(2d\right)}$. Let $z=(f_{1},\ldots ,f_{n})$ and Γ be a decision tree over *z* with the minimum depth, which solves the problem *z* and uses both attributes and hypotheses. The *d*-complete tree *G* has $2^{d}$ complete paths $\xi_{1},\ldots ,\xi_{2^{d}}$. For $i=1,\ldots ,2^{d}$, we denote by $a_{i}$ a solution of the equation system $S(\xi_{i})$. Denote $B=\{a_{1},\ldots ,a_{2^{d}}\}$. We now show that the decision tree Γ contains a complete path, the length of which is at least $\frac{d}{\log_{2}\left(2d\right)}$. We describe the process of this path construction beginning with the root of Γ.

Let the root of Γ be labeled with an attribute $f_{i_{0}}$. For δ∈{0,1}, we denote by $B^{\delta }$ the set of solutions on *B* of the equation system $\left\{f_{i_{0}}\left(x\right)=\delta \right\}$ and choose σ∈{0,1} for which $|B^{\sigma }\left|=\max\{\right|B^{0}\left|,\right|B^{1}\left|\right\}$. It is clear that $|B^{\sigma }|\geq \frac{\left|B\right|}{2}\geq \frac{\left|B\right|}{2d}$. In the considered case, the beginning of the constructed path in Γ is the root of Γ, the edge that leaves the root and is labeled with the equation system $\left\{f_{i_{0}}\left(x\right)=\sigma \right\}$, and the node to which this edge enters.

Let as assume now that the root of Γ is labeled with a hypothesis $H=\{f_{1}\left(x\right)=\delta_{1},\ldots ,f_{n}\left(x\right)=\delta_{n}\}$. We denote by $\xi_{H}$ the complete path in *G* for which the system of equations $S(\xi_{H})$ is a subsystem of *H*. Let the nonterminal nodes of the complete path $\xi_{H}$ be labeled with the attributes $f_{i_{1}},\ldots ,f_{i_{d}}$. For j=1,…,d, we denote by $B_{j}$ the set of solutions on *B* of the equation system $\left\{f_{i_{j}}\left(x\right)=\neg \delta_{i_{j}}\right\}$. It is clear that $|B_{1}\left|+\cdots +\right|B_{d}|\geq |B|-1$. Therefore, there exists l∈{1,…,d} such that $|B_{l}|\geq \frac{|B|-1}{d}\geq \frac{\left|B\right|}{2d}$. In the considered case, the beginning of the constructed path in Γ is the root of Γ, the edge that leaves the root and is labeled with the equation system $\left\{f_{i_{l}}\left(x\right)=\neg \delta_{i_{l}}\right\}$, and the node to which this edge enters.

We continue the construction of the complete path in Γ in the same way such that after the *t*th query, we have at least $\frac{\left|B\right|}{{\left(2d\right)}^{t}}$ elements from *B*. The process of path construction continues at least until $\frac{\left|B\right|}{{\left(2d\right)}^{t}}\leq 1$, i.e., at least until $\log_{2}\left|B\right|\leq t\log_{2}\left(2d\right)$. Since $\left|B\right|=2^{d},$ we have $h\left(\Gamma \right)\geq t\geq \frac{d}{\log_{2}\left(2d\right)}$ and $h_{U}^{\left(3\right)}\left(z\right)\geq \frac{d}{\log_{2}\left(2d\right)}$. □

**Lemma** **2.** *Let U=(A,F) be a binary information system, k∈N∪{0}, and U not be an m-information system for m=0,…,k. Then, there exists a (k+1)-complete tree over U.*

**Proof.**  We prove the considered statement by induction on *k*. Let k=0. In this case, *U* is not a 0-information system. Then, there exists an attribute f∈F, which is not constant on *A*. Using this attribute, it is easy to construct a 1-complete tree over *U*.

Let the considered statement hold for some *k*, k≥0. We now show that it also holds for k+1. Let U=(A,F) be a binary information system, which is not an *m*-information system for m=1,…,k+1. Then, there exists an attribute f∈F such that, for any δ∈{0,1}, the information system $U_{\delta }=\left(A\left(f,\delta \right),F\right)$ is not an *m*-information system for m=1,…,k. Using the inductive hypothesis, we conclude that, for any δ∈{0,1}, there exists a (k+1)-complete tree $G_{\delta }$ over $U_{\delta }$. Denote by *G* a directed tree with root in which the root is labeled with the attribute *f*, and for any δ∈{0,1}, there is an edge that leaves the root, is labeled with the equation system {f(x)=δ}, and enters the root of the tree $G_{\delta }$. One can show that the tree *G* is a (k+2)-complete tree over *U*. □

**Proof** **of** **Theorem 2.** It is clear that $h_{U}^{\left(3\right)}\left(z\right)\leq h_{U}^{\left(2\right)}\left(z\right)$ for any problem *z* over *U*. Therefore, $h_{U}^{\left(3\right)}\left(n\right)\leq h_{U}^{\left(2\right)}\left(n\right)$ for any n∈N.

(a) Let k∈N∪{0}. We now show by induction on *k* that, for each binary *k*-information system *U* (not necessarily infinite) for each problem *z* over *U*, the inequality $h_{U}^{\left(2\right)}\left(z\right)\leq k$ holds. Let U=(A,F) be a binary 0-information system and *z* be a problem over *U*. Since all attributes from F(z) are constant on *A*, the set $\Delta_{U}\left(z\right)$ contains only one tuple. Therefore, the decision tree containing only one node labeled with this tuple solves the problem *z* relative to *U*, and $h_{U}^{\left(2\right)}\left(z\right)=0$.

Let k∈N∪{0} and, for each *m*, 0≤m≤k, the considered statement hold. Let us show that it holds for k+1. Let U=(A,F) be a binary (k+1)-information system and $z=(f_{1},\ldots ,f_{n})$ be a problem over *U*. For i=1,…,n, choose a number $\delta_{i}\in \left\{0,1\right\}$ such that the information system $\left(A\left(f_{i},\neg \delta_{i}\right),F\right)$ is an $m_{i}$-information system where $1\leq m_{i}\leq k$. Using the inductive hypothesis, we conclude that, for i=1,…,n, there is a decision tree $\Gamma_{i}$ over *z*, which uses only hypotheses, solves the problem *z* over $\left(A\left(f_{i},\neg \delta_{i}\right),F\right)$, and has depth at most $m_{i}$. We denote by Γ a decision tree in which the root is labeled with the hypothesis $H=\{f_{1}\left(x\right)=\delta_{1},\ldots ,f_{n}\left(x\right)=\delta_{n}\}$, the edge leaving the root and labeled with *H* enters the terminal node labeled with the tuple $\left(\delta_{1},\ldots ,\delta_{n}\right)$, and for i=1,…,n, the edge leaving the root and labeled with $\left\{f_{i}\left(x\right)=\neg \delta_{i}\right\}$ enters the root of the tree $\Gamma_{i}$. One can show that Γ solves the problem *z* relative to *U* and h(Γ)≤k+1. Therefore, $h_{U}^{\left(2\right)}\left(z\right)\leq k+1$ for any problem *z* over *U*.

Let U∈C. Then, *U* is a *k*-information system for some natural *k*, and for each problem *z* over *U*, we have $h_{U}^{\left(3\right)}\left(z\right)\leq h_{U}^{\left(2\right)}\left(z\right)\leq k$. Therefore, $h_{U}^{\left(2\right)}\left(n\right)=O\left(1\right)$ and $h_{U}^{\left(3\right)}\left(n\right)=O\left(1\right)$.

(b) Let U=(A,F)∈D∖C. First, we show that $h_{U}^{\left(2\right)}\left(n\right)=O\left(\log n\right)$. Let $z=(f_{1},\ldots ,f_{n})$ be an arbitrary problem over *U*. From Lemma 5.1 [16], it follows that $|\Delta_{U}{\left(z\right)|\leq \left(4n\right)}^{I(U)}$. The proof of this lemma is based on results similar to the ones obtained by Sauer [20] and Shelah [21]. We consider a decision tree Γ over *z*, which solves *z* relative to *U* and uses only hypotheses. This tree is constructed by the halving algorithm [1,12]. We describe the work of this tree for an arbitrary element *a* from *A*. Set Δ=$\Delta_{U}\left(z\right)$. If |Δ|=1, then the only *n*-tuple from Δ is the solution z(a) of the problem *z* for the element *a*. Let |Δ|≥2. For i=1,…,m, we denote by $\delta_{i}$ a number from {0,1} such that $|\Delta \left(f_{i},\delta_{i}\right)|\geq |\Delta \left(f_{i},\neg \delta_{i}\right)|$. The root of Γ is labeled with the hypothesis $H=\{f_{1}\left(x\right)=\delta_{1},\ldots ,f_{n}\left(x\right)=\delta_{n}\}$. After this query, either the problem *z* is solved (if the answer is *H*) or we halve the number of objects in the set Δ (if the answer is a counterexample $\left\{f_{i}\left(x\right)=\neg \delta_{i}\right\}$). In the latter case, set Δ=$\Delta_{U}\left(z\right)\left(f_{i},\neg \delta_{i}\right)$. The decision tree Γ continues to work with the element *a* and the set of *n*-tuples Δ in the same way. Let, during the work with the element *a*, the considered decision tree make *q* queries. After the (q−1)th query, the number of remaining *n*-tuples in the set Δ is at least two and at most ${\left(4n\right)}^{I(U)}/2^{q-1}$. Therefore, $2^{q}\leq {\left(4n\right)}^{I(U)}$ and $q\leq I\left(U\right)\log_{2}\left(4n\right)$. Therefore, during the processing of the element *a*, the decision tree Γ makes at most $I\left(U\right)\log_{2}\left(4n\right)$ queries. Since *a* is an arbitrary element from *A*, the depth of Γ is at most $I\left(U\right)\log_{2}\left(4n\right)$. Since *z* is an arbitrary problem over *U*, we obtain $h_{U}^{\left(2\right)}\left(n\right)=O\left(\log n\right)$. Therefore, $h_{U}^{\left(3\right)}\left(n\right)=O\left(\log n\right)$.

Using Lemma 2 and the relation U∉C, we obtain that, for any d∈N, there exists *d*-complete tree $G_{d}$ over *U*. Let $F\left(G_{d}\right)=\left\{f_{1},\ldots ,f_{n_{d}}\right\}$. We know that $n_{d}\leq 2^{d}$. Denote $z_{d}=\left(f_{1},\ldots ,f_{n_{d}}\right)$. From Lemma 1, it follows that $h_{U}^{\left(2\right)}\left(z_{d}\right)\geq d$ and $h_{U}^{\left(3\right)}\left(z_{d}\right)\geq \frac{d}{\log_{2}\left(2d\right)}$. As a result, we have $h_{U}^{\left(2\right)}\left(2^{d}\right)\geq d$ and $h_{U}^{\left(3\right)}\left(2^{d}\right)\geq \frac{d}{\log_{2}\left(2d\right)}$. Let n∈N and n≥8. Then, there exists d∈N such that $2^{d}\leq n<2^{d+1}$. We have $d>\log_{2}n-1$, $h_{U}^{\left(2\right)}\left(n\right)\geq \log_{2}n-1$, $h_{U}^{\left(2\right)}\left(n\right)=\Omega \left(\log n\right)$, and $h_{U}^{\left(2\right)}\left(n\right)=\Theta \left(\log n\right)$. It is easy to show that the function $\frac{x}{\log_{2}\left(2x\right)}$ is nondecreasing for x≥2. Therefore, $h_{U}^{\left(3\right)}\left(n\right)\geq \frac{\log_{2}n-1}{\log_{2}\left(2\left(\log_{2}n-1\right)\right)}$ and $h_{U}^{\left(3\right)}\left(n\right)=\Omega \left(\frac{\log n}{\log\log n}\right)$.

(c) Let U=(A,F)∉D. We now consider an arbitrary problem $z=(f_{1},\ldots ,f_{n})$ over *U* and a decision tree over *z*, which uses only hypotheses and solves the problem *z* over *U* in the following way. For a given element a∈A, the first query is about the hypothesis $H_{1}=\left\{f_{1}\left(x\right)=1,\ldots ,f_{n}\left(x\right)=1\right\}$. If the answer is $H_{1}$, then the problem *z* is solved for the element *a*. If, for some i∈{1,…,n}, the answer is $\left\{f_{i}\left(x\right)=0\right\}$, then the second query is about the hypothesis $H_{2}$ obtained from $H_{1}$ by replacing the equality $f_{i}\left(x\right)=1$ with the equality $f_{i}\left(x\right)=0$, etc. It is clear that after at most *n* queries, the problem *z* for the element *a* will be solved. Thus, $h_{U}^{\left(2\right)}\left(z\right)\leq n$ and $h_{U}^{\left(3\right)}\left(z\right)\leq n$. Since *z* is an arbitrary problem over *U*, we have $h_{U}^{\left(2\right)}\left(n\right)\leq n$ and $h_{U}^{\left(3\right)}\left(n\right)\leq n$ for any n∈N.

Let n∈N. Since U∉D, there exist attributes $f_{1},\ldots ,f_{n}\in F$ such that, for any $\left(\delta_{1},\ldots ,\delta_{n}\right)\in {\left\{0,1\right\}}^{n}$, the equation system $\left\{f_{1}\left(x\right)=\delta_{1},\ldots ,f_{n}\left(x\right)=\delta_{n}\right\}$ is consistent on *A*. We now consider the problem $z=(f_{1},\ldots ,f_{n})$ and an arbitrary decision tree Γ over *z*, which solves the problem *z* over *U* and uses both attributes and hypotheses. Let us show that h(Γ)≥n. If n=1, then the considered inequality holds since $|\Delta_{U}\left(z\right)|\geq 2$. Let n≥2. It is easy to show that an equation system over *z* is inconsistent if and only if it contains equations $f_{i}\left(x\right)=0$ and $f_{i}\left(x\right)=1$ for some i∈{1,…,n}. For each node *v* of the decision tree Γ, we denote by $S_{v}$ the union of systems of equations attached to edges in the path from the root of Γ to *v*. A node *v* of Γ will be called consistent if the equation system $S_{v}$ is consistent.

We now construct a complete path ξ in the decision tree Γ, for which the nodes are consistent. We start from the root that is a consistent node. Let the path reach a consistent node *v* of Γ. If *v* is a terminal node, then the path ξ is constructed. Let *v* be a working node labeled with an attribute $f_{i}\in F\left(z\right)$. Then, there exists δ∈{0,1} for which the system of equations $S_{v}\cup \left\{f_{i}\left(x\right)=\delta \right\}$ is consistent. Then, the path ξ will pass through the edge leaving *v* and labeled with the system of equations $\left\{f_{i}\left(x\right)=\delta \right\}$. Let *v* be labeled with a hypothesis $H=\{f_{1}\left(x\right)=\delta_{1},\ldots ,f_{n}\left(x\right)=\delta_{n}\}$. If there exists i∈{1,…,n} such that the system of equations $S_{v}\cup \left\{f_{i}\left(x\right)=\neg \delta \right\}$ is consistent, then the path ξ will pass through the edge leaving *v* and labeled with the system of equations $\left\{f_{i}\left(x\right)=\neg \delta \right\}$. Otherwise, $S_{v}=H$, and the path ξ will pass through the edge leaving *v* and labeled with the system of equations *H*.

Let all edges in the path ξ be labeled with systems of equations containing one equation each. Since all nodes of ξ are consistent, the equation system S(ξ) is consistent. We now show that S(ξ) contains at least *n* equations. Let us assume that this system contains less than *n* equations. Then, the set $\Delta_{U}\left(z\right)\pi \left(\xi \right)$ contains more than one *n*-tuple, which is impossible. Therefore, the length of the path ξ is at least *n*. Let there be edges in ξ, which are labeled with hypotheses, and the first edge in ξ labeled with a hypothesis *H* leaves the node *v*. Then, $S_{v}=H$, and the length of ξ is at least *n*. Therefore, h(Γ)≥n, $h_{U}^{\left(3\right)}\left(z\right)\geq n$, and $h_{U}^{\left(2\right)}\left(z\right)\geq n$. As a result, we obtain $h_{U}^{\left(3\right)}\left(n\right)\geq n$ and $h_{U}^{\left(2\right)}\left(n\right)\geq n$. Thus, $h_{U}^{\left(2\right)}\left(n\right)=n$ and $h_{U}^{\left(3\right)}\left(n\right)=n$ for any n∈N. □

## 5. Proof of Theorem 3

First, we prove several auxiliary statements.

**Proposition** **1.** *R⊆D.*

**Proof.**  Let U∈R. By Theorem 1, $h_{U}^{\left(1\right)}\left(n\right)=\Theta \left(\log n\right)$. Let us assume that U∉D. Then, for any n∈N, there exists a problem $z=(f_{1},\ldots ,f_{n})$ over *U* such that $|\Delta_{U}\left(z\right)|=2^{n}$. Let Γ be a decision tree over *z*, which solves the problem *z* relative to *U* and uses only attributes. Then, Γ should have at least $2^{n}$ terminal nodes. One can show that the number of terminal nodes in the tree Γ is at most $2^{h(\Gamma )}$. Then, $2^{n}\leq 2^{h(\Gamma )}$, h(Γ)≥n, and $h_{U}\left(z\right)\geq$n. Therefore, $h_{U}^{\left(1\right)}\left(n\right)\geq n$ for any n∈N, which is impossible. Thus, R⊆D. □

**Proposition** **2.** *C⊆D.*

**Proof.**  Let U∈C. By Theorem 2, $h_{U}^{\left(2\right)}\left(n\right)=O\left(1\right)$. Let us assume that U∉D. Then, by Theorem 2, $h_{U}^{\left(2\right)}\left(n\right)=n$ for any n∈N, which is impossible. Therefore, C⊆D. □

**Proposition** **3.** *R∩C=∅.*

**Proof.**  Assume the contrary: R∩C≠∅ and U=(A,F)∈R∩C. Let r,k∈N, *U* be an *r*-reduced information system and *U* be a *k*-information system. We now consider an arbitrary problem $z=(f_{1},\ldots ,f_{n})$ over *U* and describe a decision tree Γ over *z*, which uses only attributes, solves the problem *z* over *U*, and has depth at most kr.

For i=1,…,n, let $\delta_{i}$ be a number from {0,1} such that $\left(A\left(f_{i},\neg \delta_{i}\right),F\right)$ is an $m_{i}$-information system with $0\leq m_{i}<k$. Let *t* be the maximum number from the set {1,…,n} such that the system of equations $S=\{f_{1}\left(x\right)=\delta_{1},\ldots ,f_{t}\left(x\right)=\delta_{t}\}$ is consistent. Then, there exists a subsystem $\left\{f_{i_{1}}\left(x\right)=\delta_{i_{1}},\ldots ,f_{i_{p}}\left(x\right)=\delta_{i_{p}}\right\}$ of the system *S*, which has the same set of solutions as *S* and for which p≤r. For a given a∈A, the decision tree Γ computes sequentially values $f_{i_{1}}\left(a\right),\ldots ,f_{i_{p}}\left(a\right)$.

If, for some q∈{1,…,p}, $f_{i_{1}}\left(a\right)=\delta_{i_{1}},\ldots ,f_{i_{q-1}}\left(a\right)=\delta_{i_{q-1}}$, and $f_{i_{q}}\left(a\right)=\neg \delta_{i_{q}}$, then the decision tree Γ continues to work with the problem *z* and the information system $U^{\prime }=\left(A^{\prime },F\right)$ where $A^{\prime }$ is the set of solutions on *A* of the equation system $\left\{f_{i_{1}}\left(x\right)=\delta_{i_{1}},\ldots ,f_{i_{q-1}}\left(x\right)=\delta_{i_{q-1}},f_{i_{q}}\left(x\right)=\neg \delta_{i_{q}}\right\}$. We have that $U^{\prime }$ is an $l^{\prime }$-information system for some $l^{\prime }\leq m_{i_{q}}<k$.

Let $f_{i_{1}}\left(a\right)=\delta_{i_{1}},\ldots ,f_{i_{p}}\left(a\right)=\delta_{i_{p}}$. If t=n, then $\left(\delta_{1},\ldots ,\delta_{n}\right)$ is the solution of the problem *z* for the considered element *a*. Let t<n. Then, the decision tree Γ continues to work with the problem *z* and the information system $U^{\prime \prime }=\left(A^{\prime \prime },F\right)$ where $A^{\prime \prime }$ is the set of solutions on *A* of the equation system $\left\{f_{i_{1}}\left(x\right)=\delta_{i_{1}},\ldots ,f_{i_{p}}\left(x\right)=\delta_{i_{p}}\right\}$. We know that the equation system $\left\{f_{1}\left(x\right)=\delta_{1},\ldots ,f_{t}\left(x\right)=\delta_{t},f_{t+1}\left(x\right)=\delta_{t+1}\right\}$ is inconsistent. Therefore, the system $\left\{f_{i_{1}}\left(x\right)=\delta_{i_{1}},\ldots ,f_{i_{p}}\left(x\right)=\delta_{i_{p}},f_{t+1}\left(x\right)=\delta_{t+1}\right\}$ is inconsistent. Hence, $A^{\prime \prime }\subseteq A\left(f_{t+1},\neg \delta_{t+1}\right)$ and $U^{\prime \prime }$ is an $l^{\prime \prime }$-information system for some $l^{\prime \prime }\leq m_{t+1}<k$.

As a result, after the computation of the values of at most *r* attributes, we either solve the problem *z* or reduce the consideration of the problem *z* over the *k*-information system *U* to the consideration of the problem *z* over some *l*-information system where l<k. After the computation of the values of at most rk attributes, we solve the problem *z* since each problem over the 0-information system has exactly one possible solution. Therefore, $h_{U}^{\left(1\right)}\left(z\right)\leq rk$ and $h_{U}^{\left(1\right)}\left(n\right)=O\left(1\right)$. By Theorem 1, $h_{U}^{\left(1\right)}\left(n\right)=\Theta \left(\log n\right)$. The obtained contradiction shows that R∩C=∅. □

**Proposition** **4.** *For any infinite binary information system, its indicator vector coincides with one of the rows of Table 1.*

**Proof.**  Table 3 contains as rows all three-tuples from the set ${\left\{0,1\right\}}^{3}$. We now show that the rows with the numbers 5–8 cannot be indicator vectors of infinite binary information systems. Assume the contrary: there is i∈{5,6,7,8} such that the row with the number *i* is the indicator vector of an infinite binary information system *U*. If i=5, then U∈R and U∉D, but this is impossible, since, by Proposition 1, R⊆D. If i=6, then U∈C and U∉D, but this is impossible, since, by Proposition 2, C⊆D. If i=7, then U∈R and U∉D, but this is impossible, since, by Proposition 1, R⊆D. If i=8, then U∈R and U∈C, but this is impossible, since, by Proposition 3, R∩C=∅. Therefore, for any infinite binary information system, its indicator vector coincides with one of the rows of Table 3 with Numbers 1–4. Thus, it coincides with one of the rows of Table 1. □

Define an infinite binary information system $U_{1}=\left(A_{1},F_{1}\right)$ as follows: $A_{1}=N$ and $F_{1}$ is the set of all functions from N to {0,1}.

**Lemma** **3.** *The information system $U_{1}$ belongs to the class $V_{1}$.*

**Proof.**  It is easy to show that the information system $U_{1}$ has an infinite *I*-dimension. Therefore, $U_{1}\notin D$. Using Proposition 4, we obtain ind(U)=(0,0,0), i.e., $U_{1}\in V_{1}$. □

For any i∈N, we define two functions $p_{i}:N\to \left\{0,1\right\}$ and $l_{i}:N\to \left\{0,1\right\}$. Let j∈N. Then, $p_{i}\left(j\right)=1$ if and only if j=i and $l_{i}\left(j\right)=1$ if and only if j>i.

Define an infinite binary information system $U_{2}=\left(A_{2},F_{2}\right)$ as follows: $A_{2}=N$ and $F_{2}=\left\{p_{i}:i\in N\right\}\cup \left\{l_{i}:i\in N\right\}$.

**Lemma** **4.** *The information system $U_{2}$ belongs to the class $V_{2}$.*

**Proof.**  For n∈N, denote $S_{n}=\left\{p_{1}\left(x\right)=0,\ldots ,p_{n}\left(x\right)=0\right\}$. One can show that the equation system $S_{n}$ is consistent and each proper subsystem of $S_{n}$ has a set of solutions different from the set of solutions of $S_{n}$. Therefore, $U_{2}\notin R$. Using attributes from the set $\left\{l_{i}:i\in N\right\}$, we can construct a *d*-complete tree over $U_{2}$ for each d∈N. By Lemma 1 and Theorem 2, $U_{2}\notin C$. One can show that $I(U_{2})=1$. Therefore, $U_{2}\in D$. Thus, $ind\left(U_{2}\right)=\left(0,1,0\right)$, i.e., $U_{2}\in V_{2}$. □

Define an infinite binary information system $U_{3}=\left(A_{3},F_{3}\right)$ as follows: $A_{3}=N$ and $F_{3}=\left\{p_{i}:i\in N\right\}$.

**Lemma** **5.** *The information system $U_{3}$ belongs to the class $V_{3}$.*

**Proof.**  It is easy to show that $U_{3}$ is a 1-information system. Therefore, $U_{3}\in C$. Using Proposition 4, we obtain $ind\left(U_{3}\right)=\left(0,1,1\right)$, i.e., $U_{3}\in V_{3}$. □

Define an infinite binary information system $U_{4}=\left(A_{4},F_{4}\right)$ as follows: $A_{4}=N$ and $F_{4}=\left\{l_{i}:i\in N\right\}$.

**Lemma** **6.** *The information system $U_{4}$ belongs to the class $V_{4}$.*

**Proof.**  Let us consider an arbitrary consistent system of equations *S* over $U_{4}$. We now show that there is a subsystem of *S*, which has at most two equations and the same set of solutions as *S*. Let *S* contain both equations of the kind $l_{i}\left(x\right)=1$ and $l_{j}\left(x\right)=0$. Denote $i_{0}=\max\left\{i:l_{i}\left(x\right)=1\in S\right\}$ and $j_{0}=\min\left\{j:l_{j}\left(x\right)=0\in S\right\}$. One can show that the system of equations $S^{\prime }=\left\{l_{i_{0}}\left(x\right)=1,l_{j_{0}}\left(x\right)=0\right\}$ has the same set of solutions as *S*. The case when *S* contains for some δ∈{0,1} only equations of the kind $l_{p}\left(x\right)=\delta$ can be considered in a similar way. In this case, the equation system $S^{\prime }$ contains only one equation. Therefore, the information system $U_{4}$ is 2-reduced and $U_{4}\in$R. Using Proposition 4, we obtain $ind\left(U_{4}\right)=\left(1,1,0\right)$, i.e., $U_{4}\in V_{4}$. □

**Proof** **of** **Theorem 3.** From Proposition 4, it follows that, for any infinite binary information system, its indicator vector coincides with one of the rows of Table 1. Using Lemmas 3–6, we conclude that each row of Table 1 is the indicator vector of some infinite binary information system. □

## 6. Conclusions

Based on the results of exact learning, test theory, and rough set theory, for an arbitrary infinite binary information system, we studied three functions of the Shannon type, which characterize the dependence in the worst case of the minimum depth of a decision tree solving a problem on the number of attributes in the problem description. These three functions correspond to (i) decision trees using attributes, (ii) decision trees using hypotheses, and (iii) decision trees using both attributes and hypotheses. We described possible types of behavior for each of these three functions. We also studied the join behavior of these functions and distinguished four corresponding complexity classes of infinite binary information systems. In the future, we plan to translate the obtained results into the language of exact learning.

The problems studied in this paper allow us to confine ourselves to considering only the crisp (conventional) sets that are completely defined by attributes. However, in the future, when we investigate approximately defined problems or approximate decision trees, it will be necessary to work with rough sets given by their lower and upper approximations. This will require a wider range of rough set theory techniques than those used in the present paper.

## Figures and Tables

**Table 1:** Possible indicator vectors of infinite binary information systems.

	R	D	C
1	0	0	0
2	0	1	0
3	0	1	1
4	1	1	0

**Table 2:** Summary of Theorems 1–3.

	R	D	C	$h_{U}^{\left(1\right)}\left(n\right)$	$h_{U}^{\left(2\right)}\left(n\right)$	$h_{U}^{\left(3\right)}\left(n\right)$
$V_{1}$	0	0	0	*n*	*n*	*n*
$V_{2}$	0	1	0	*n*	Θ(logn)	$\Omega \left(\frac{\log n}{\log\log n}\right),O\left(\log n\right)$
$V_{3}$	0	1	1	*n*	O(1)	O(1)
$V_{4}$	1	1	0	Θ(logn)	Θ(logn)	$\Omega \left(\frac{\log n}{\log\log n}\right),O\left(\log n\right)$

**Table 3:** All 3-tuples from the set ${\left\{0,1\right\}}^{3}$.

	R	D	C
1	0	0	0
2	0	1	0
3	0	1	1
4	1	1	0
5	1	0	0
6	0	0	1
7	1	0	1
8	1	1	1

## Data Availability

Not applicable.
